# Biomechanics of Fastpitch Softball Pitching: A Practitioner’s Guide

**DOI:** 10.1177/19417381251323610

**Published:** 2025-04-03

**Authors:** Kenzie B. Friesen, Lauren S. Butler, Nicole M. Bordelon, Jessica L. Downs-Talmage, Glenn S. Fleisig, Sophia Ulman, Gretchen D. Oliver

**Affiliations:** † University of Saskatchewan, Saskatchewan, Canada; ‡ Florida International University, Florida; § Nicklaus Children’s Hospital, Florida; ‖ Yale University, Connecticut; ¶ Northern State University, Aberdeen, South Dakota; # American Sports Medicine Institute, Birmingham, Alabama; †† Scottish Rite for Children, Frisco, Texas; ‡‡ University of Texas Southwestern Medical Center, Dallas, Texas; §§ Auburn University, Auburn, Alabama

**Keywords:** biomechanics, injury, kinematics, training, windmill

## Abstract

**Context:**

Despite fastpitch softball’s growing popularity, there is limited evidence-based guidance to aid practitioners in developing pitching-specific injury prevention and performance enhancement strategies. This commentary describes the biomechanics across each phase of the softball pitch and provides explanation of common biomechanical errors during the pitch as well as training strategies and exercise recommendations to foster optimal pitcher development.

**Evidence Acquisition:**

A review of softball pitching biomechanics research available in electronic databases including PubMed, Medline, and EBSCO.

**Study Design:**

Clinical review.

**Level of Evidence:**

Level 4.

**Results:**

The 4 primary phases of the windmill softball pitch include the wind-up, stride, acceleration, and follow-through.

**Conclusion:**

Specific training strategies are recommended to combat the various flaws associated with each phase of the softball pitch. Evaluating body composition, functional characteristics like strength and range of motion of the shoulders, trunk, and hips, as well as assessing energy flow may result in improved performance and minimize risk of injury.

Fastpitch softball is one of the most popular sports among North American youth and collegiate athletes.^33,55^ One of the notable aspects of this sport is the windmill softball pitch—a complex movement requiring coordination of the kinetic chain and complete throwing shoulder circumduction in an underhand motion.^3,60^ Although the underhand motion is often perceived as less demanding on the upper extremity compared with overhand throwing, softball pitchers experience shoulder distraction forces near 100% of bodyweight,^3^ and high rates of upper extremity pain and overuse injury.^32,50^ Specifically, pitchers are 2.6 times more likely to sustain an upper extremity injury compared with positional players.^54^ Further, high practice and in-game pitch volumes are frequently accumulated due to teams relying on small pitching rosters. Therefore, athletes, coaches, and clinicians need to understand the best practices of softball pitching.

Due to high participation and overuse injury rates, there has been an increase in research dedicated to understanding the biomechanical implications of the softball pitch.^50^ Emerging literature has considered various components related to overuse injury, performance, anthropometrics, functional characteristics, kinetics, and kinematic parameters across various age groups and levels of competition, energy flow descriptions, and clinical commentaries.^4,13,14,19,24-26,29,38^ Given the recent surge in softball research, it is pertinent to synthesize the current literature to provide a guide of current evidence-based findings for performance and safety among fastpitch softball pitchers.

The main objective of this paper is to describe the biomechanics across each phase of the softball pitch. The secondary objective is to educate health practitioners, coaches, and athletes on common biomechanical errors during the pitch as well as provide training strategies and exercise recommendations to foster optimal pitcher development. The third objective is to present a discussion of the recent literature surrounding pitch type considerations, energy flow, functional characteristics, and anthropometrics for pitchers.

## Biomechanics of the Windmill Softball Pitch

**Figure 1.**
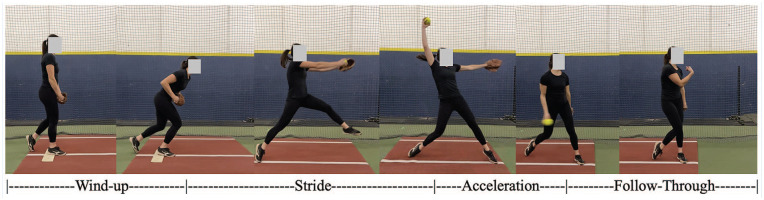
The 4 phases of the windmill pitch.

The full-body dynamic movement of the windmill softball pitch is broken down into the following 4 phases: (1) wind-up, (2) stride, (3) acceleration, and (4) follow-through (Figure 1). The sections below provide key biomechanical descriptions of each pitch phase.

### Wind-up Phase

The softball pitch starts at the onset of movement and ends as the stride foot toe leaves the ground (Figure 1).^3^ The goal of this phase is to position the athlete for a powerful drive off the pitching mound. The optimal wind-up phase of the pitch begins with a posterior weight shift onto the stride leg (contralateral to the pitching arm) to initiate momentum.^26,39^ This shift requires considerable activation of the gluteal muscle group, especially the gluteus maximus.^26,48^ During the wind-up, throwing and glove arm mechanics can vary depending on the pitcher’s preferred style,^41,60^ and may involve a backward swing of both arms, only the pitching arm, or neither. If neither, the arms remain together with the ball in the glove during the wind-up. Regardless of arm swing variation, pitchers should position themselves in a way that allows them to generate optimal force in the plane of the pitch. Given the parallels between the force production and power needed in both sprinting activities and driving forcefully off the pitching mound for optimal pitch velocity, pitchers are encouraged to assume a “sprinter-like position” with both knees flexed, the heel of the back foot off the ground, a slight forward trunk lean, and the body squared to home plate (Table 1, entry A) at the end of the wind-up phase. This position allows the pitcher to produce a powerful forward push down the mound during the stride phase. Research shows the amount of force generated during this phase relates to pitch velocity,^39^ highlighting the importance of proper positioning during the wind-up in preparation for the stride.

**Table 1.** Assessment per pitch phase

Variable	Wind-up Phase	
	Assessment	
	Description	Image
A. Sagittal alignment	At the end of the wind-up phase, draw a line down the trunk and a separate line down the tibia. The trunk line and tibia line should remain roughly parallel	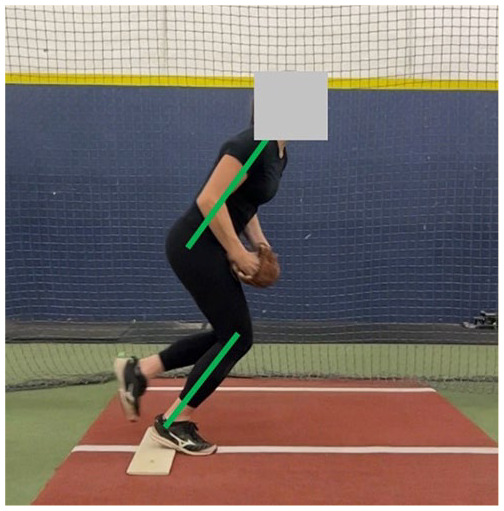
B. Frontal plane trunk position	At the end of the wind-up phase, the hips should be relatively square to home plate	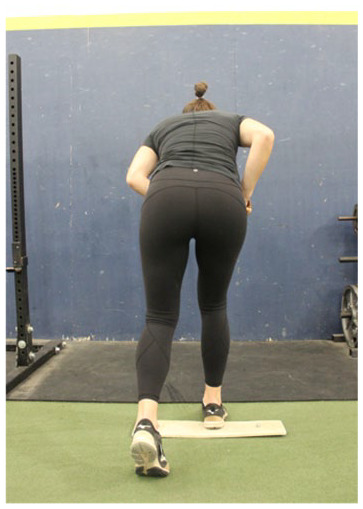
C. Frontal plane knee alignment	At the end of the wind-up phase, draw a vertical line down from the knee joint center on the drive leg to the heel. The line should fall in line with the middle of the drive foot	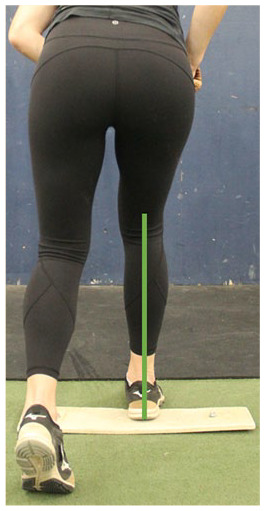
D. Wind-up phase foot position	At the end of the wind-up phase, the drive foot should be pointed toward home plate and placed directly on the power line	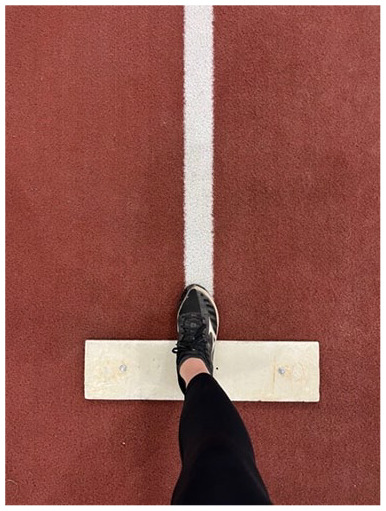
E. Sagittal trunk and hip alignment	At the start of stride, the drive leg should undergo a forceful triple extension and push away from the mound. The shoulders and trunk should remain facing home plate and the shoulders and stride leg hip should also be flexed toward home plate	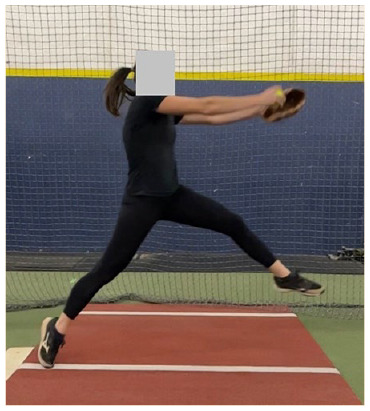
F. Foot position at SFC	At SFC, measure the angle formed between a line drawn from the mid ankle through the foot and a line from the mid ankle to home plate. The angle should be between 0 and 45° toward the pitching arm side	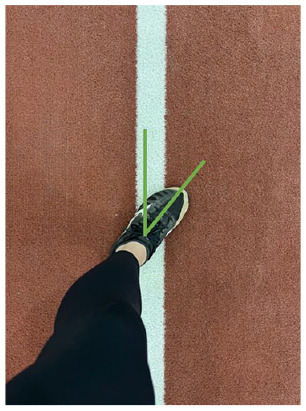
G. Arm path at SFC	At SFC, observe the pitching arm relative to the power line. The arm should remain relatively perpendicular to the ground and be close to the power line	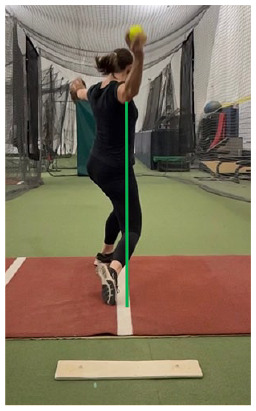
H. Sagittal trunk alignment at SFC	At SFC, draw a vertical line, which should pass through the belly button, from the head to the ground. The drag should occur on the inside of the big toe	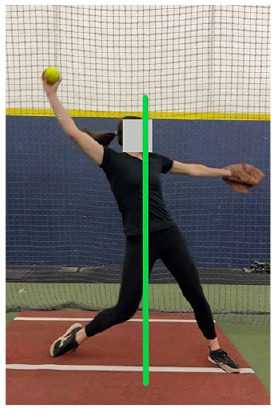
I. Frontal plane knee alignment at SFC	At SFC, draw a vertical line from the knee joint center to the ground. The line should fall roughly in line with the ankle joint center	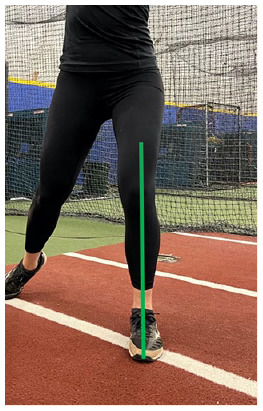
J. Hip and shoulder position	At SFC, the pelvis and trunk should be rotated toward the pitching arm side	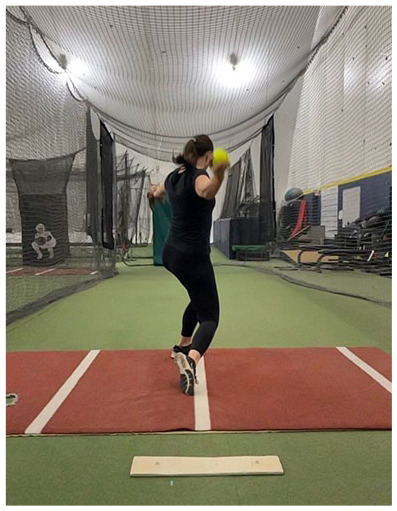
	Acceleration Phase	
	Assessment	
	Description	Image
K. Acceleration arm path	During arm acceleration, the path of the arm should remain close to the body and the back leg should remain close to the power line	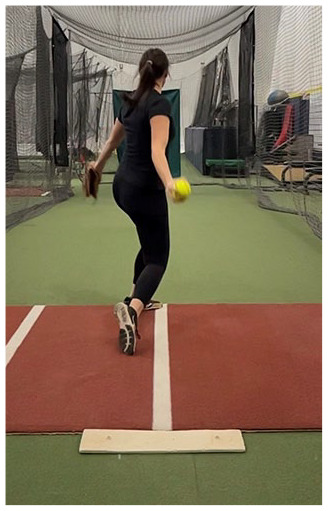
	Follow Through Phase	
	Assessment	
	Description	Image
L. Single leg stability at follow through	At the end of the follow-through phase, draw a vertical line down from the knee joint center to the ground. The line should fall roughly in line with the ankle joint center	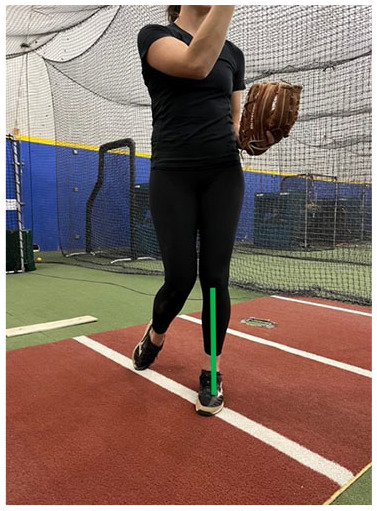

SFC, stride foot contact.

### Assessment and Training Strategies

#### Fault: Downward Shift of Bodyweight

A common fault during the wind-up phase involves the pitcher shifting their bodyweight downward via increased knee flexion, instead of performing a hip hinge and shifting weight backward. This downward transfer of momentum inhibits a pitcher’s ability to drive toward home plate. Subsequently, the pitcher may jump off the mound producing more vertical ground-reaction force rather than propulsive force, which can reduce their stride length and have negative implications on performance as research highlights the importance of ground-reaction force in line with pitched ball velocity.^26^

#### Fault: Excessive Trunk Flexion or Extension

Clinical examination of the sagittal plane can be performed at the end of the wind-up phase by assessing the trunk and drive leg position. Optimal sagittal alignment is identified when the trunk remains roughly parallel to the tibia (Table 1, entry A). Suboptimal alignment is identified when the trunk moves into too much flexion or extension, resulting in a trunk position that is not parallel to the tibia. To address poor sagittal alignment, drills that encourage a quick drop into a split stance athletic position are recommended. These drills may include split stance medicine ball overhead slams, drop to split stance drills, and rear foot elevated split squats (Table 2, entry A). Of note, it is important to first perform these exercises correctly, and then progress to performing them with increased speed. We recommend starting with good form before progressing to quicker movements and ideally quicker movements can be performed so that there is better transferability of exercises to aid the fast and powerful performance of the windmill pitch.

**Table 2.** Training recommendations per pitching fault

Load Phase	
Training Recommendations	
Pitching Fault	Exercises/Drills
A. Poor sagittal trunk alignment	• Split stance medicine ball overhead slams • Quick drop to split stance drills • Rapid tempo rear foot elevated split squats
B. Excessive trunk rotation	• Trunk banded single leg squats • Single leg bridges • Super clams
C. Poor frontal plane knee alignment	• Knee banded single leg squats • Knee banded plyometric training • Lateral step downs
Stride Phase	
Training Recommendations	
Pitching Fault	Exercises/Drills
D. Excessive trunk or hip flexion	• Wall knee drives • Sled pushes • Single leg landmine drives • Rapid hip extension with neutral pelvis • Quadruped hip extension with neutral lumbar spine
E. Poor arm path at SFC	• Banded Ws • Suspended rows • Y lift offs • Closed chain serratus punches
F. Poor sagittal trunk position at SFC	• Hip thrusters • Planks • Single leg squats
G. Poor frontal plane knee position at SFC	See exercises and drills for pitching fault C.
H. Insufficient pelvis and trunk rotation	• Thread the needle • Book openers
Arm Acceleration	
Training Recommendations	
Pitching Fault	Exercises/Drills
I. Poor arm path during acceleration	• Push ups • Serratus floor slides • See exercises and drills for pitching fault *E*
J. Poor back leg position during acceleration	• Anti-rotation single leg squats • Rotational hops
Follow-Through	
Training Recommendations	
Pitching Fault	Exercises/Drills
K. Poor deceleration	• Eccentric side lying external rotation • Suspended eccentric Ys • Eccentric banded horizontal abduction
L. Single leg stability	• Single leg squats • Single leg Romanian deadlift with trunk rotation

Videos available in Online Supplemental material. SFC, stride foot contact.

#### Fault: Neutral Malignment

Common frontal plane faults during the wind-up phase include a loss of neutral alignment in the frontal plane at the trunk or knee, as well as excessive arm abduction and drive foot external rotation. The pitcher should end the wind-up with their hips and shoulders mostly square to home plate (Table 1, entry B) to allow for the most force generation during stride.^29^ A common fault is for the pitcher to rotate their hips and shoulders toward the pitching arm before striding, which may produce a lateral ground-reaction force inhibiting forward transfer of momentum.^26^ Trunk proprioception training and hip strengthening are recommended to address frontal plane stability. Specifically, drills such as banded trunk single leg squats may be used to train neutral trunk alignment. Single leg bridges and super clams may be used to strengthen the hip musculature (Table 2, entry B).

At the end of the wind-up phase, the glove and pitching arm should not inhibit a pitcher’s ability to drive squarely off the mound. Both arms should be of relatively equal height to not influence the pitcher’s trunk rotation. If a backswing of the pitching arm results in excessive trunk rotation toward the pitching arm side, it can cause the hips and trunk to rotate open toward the pitching arm early during stride. Again, the arm path should not deter the pitcher from keeping shoulders and hips relatively square toward home plate. Adequate trunk and pelvis control are necessary to maintain this position. Therefore, trunk proprioception and hip strengthening are recommended. Adequate flexibility in the pectoralis major muscle is also important to ensure the pitcher’s ability to dissociate shoulder extension and abduction from trunk rotation. If the pitcher cannot correct their trunk posture with trunk and hip targeted exercises, an assessment of pectoralis flexibility is recommended.

#### Fault: Drive Knee Valgus

Maintaining neutral knee alignment during the wind-up phase is critical to generating a powerful stride just as maintaining neutral knee alignment is critical to generating force and higher peak jump height during a counter movement vertical jump.^30^ Frontal plane knee alignment can be assessed by examining the position of the knee joint center relative to the ankle and foot. The knee should ideally be positioned directly over the foot (Table 1, entry C). If the knee tracks medially into a valgus position during the posterior weight shift to the drive leg, the knee joint is subject to higher medial loading.^36^ Given the comparisons that have previously been made between pathomechanics during the pitch and the single leg squat, a single leg squat test may also be used to assess both frontal plane knee and trunk alignment.^28^ Neuromuscular training exercises can facilitate frontal plane knee alignment, including knee banded single leg squats, knee banded plyometric training, and lateral step downs with visual cues for neutral knee alignment (Table 2, entry C).

#### Fault: Foot Malignment

At the end of the wind-up phase, the foot should be positioned toward home plate. Pitchers should ensure their foot is aligned on a straight line (often referred to as the “power line”) between the pitching rubber and home plate (Table 1, entry D). Proper foot position can be assessed by examining how the foot aligns with the power line or by assessing the global foot angle created by drawing a line from the ankle joint center to home plate.^11^ This global foot angle should ideally be close to 0°. To train proper foot position, a visual cue can be used, such as a line drawn in the dirt from the rubber to home plate (Table 1, entry D).

### Stride Phase

The stride phase begins with the drive leg in single leg stance as the stride leg lifts to stride toward home plate and ends with stride foot contact (SFC).^3^ This phase requires adequate stability to control the power and high velocity movement of the pitch. Reports indicate the lumbopelvic hip complex and the muscles surrounding the scapula work to stabilize core segments necessary to properly position the body for the acceleration phase.^48^

The ground-reaction force exerted during this phase relates to pitch velocity^39^; therefore, a strong and powerful push off the mound is imperative. The direction of force generation is also important. It is ideal to produce force in the sagittal plane to cohesively propel the body toward home plate.^26^ During the drive stage of the stride, the pitcher requires a large contraction of the gluteus maximus muscle on the drive leg.^47^ During the drive off the mound, pitchers should achieve considerable triple extension of the drive leg hip, knee, and ankle (ie, plantarflexion). The shoulders and trunk should remain predominantly square toward home plate, while the shoulders and stride leg hip should be flexed in the direction of home plate (Table 1, entry E).

Stride length receives much attention as it can vary widely among pitchers (see normative data; Table 3). Research shows the benefits and disadvantages to specific stride lengths, where a middle ground is likely the best approach. Too long of a stride can limit range of motion through the trunk, hinder the acceleration phase of the pitch, and is related to pain in pitchers.^40^ Not striding far enough may indicate insufficient force production while driving off the mound. Length of stride can also impact the body’s center of mass position. Too far of a stride is linked with increased shoulder distraction force,^42^ and may also lead to a posteriorly shifted center of mass and “anchoring” of the back foot on the ground through the pitch. This anchoring can create a braking effect counter-productive to creating a high velocity pitch. The anchor may also interfere with pitch performance, where balance over the front leg is most pertinent during pitches such as the drop ball.

**Table 3.** Normative kinematics per age group

Variable	Youth	Collegiate
Top of pitch		
Stride knee flex/extension angle, deg	22 ± 11^15^	21 ± 10^15^
Drive knee flexion/extension, deg	25 ± 18^15^	38 ± 18^15^
Stride knee flexion/extension velocity, deg/s	36 ± 190^15^	−7 ± 170^15^
Drive knee flexion/extension velocity, deg/s	−7 ± 122^15^	−12.31 ± 220^15^
Trunk flexion, deg	1 ± 18^49^	10 ± 13^21^
Trunk rotation, deg	62 ±32^49^	68 ± 16^21^
Trunk lateral flexion, deg	−3 ± 15^49^	12 ± 13^21^
Pelvic rotation, deg	76 ± 12^49^	
Stride length, %	98 ± 17^15^	93 ± 14^15^
Foot Contact		
Stride knee flex/extension angle, deg	25 ± 11^15^	22 ± 8^15^
Drive knee flexion/extension, deg	23 ± 16^15^	32 ± 15^15^
Stride knee flexion/extension velocity, deg/s	86 ± 114^15^	59 ± 131^15^
Drive knee flexion/extension velocity, deg/s	−53 ± 186^15^	−158 ± 190^15^
Trunk flexion, deg	68 ± 17^49^	17 ± 16^21^
Trunk rotation, deg	69 ± 33^49^	76 ± 18^21^
Trunk lateral flexion, deg	5 ± 13^49^	14 ±11^21^
Pelvic rotation, deg	75 ± 11^49^	
Stride length, %	89 ± 13^15^	89 ± 12^15^
Ball Release		
Stride knee flex/extension, deg	26 ± 10^15^	27 ± 9^15^
Drive knee flexion/extension, deg	32 ± 19^15^	36 ± 21^15^
Stride knee flexion/extension velocity, deg/s	−82 ± 152^15^	−173 ± 132^15^
Drive knee flexion/extension velocity, deg/s	278 ± 160^15^	212 ± 162^15^
Trunk flexion, deg	−1 ± 12^49^	18 ± 10^21^
Trunk rotation, deg	30 ± 28^49^	47 ± 27^21^
Trunk lateral flexion, deg	12 ± 9^49^	15 ± 21^21^
Pelvic rotation, deg	44 ± 12^49^	
Stride length (%)	68 ± 14^15^	73 ± 11^15^

Values are reported as mean ± SD. Trunk flexion reported as (+); trunk extension reported as (−). Trunk rotation: (+) rotation toward pitching arm side, (−) rotation toward glove arm side. Trunk lateral flexion: (=) flexion toward pitching arm side, (−) flexion toward glove arm side. Pelvis rotation: (+) rotation towards the pitching arm side, (-) rotation towards the glove arm side. Stride length is presented as a percentage of body height.

### Assessment and Training Strategies

A powerful stride depends on a strong push off the pitching rubber. Advantageous positions for optimal power production rely on the drive leg completing rapid hip extension, knee extension, and plantarflexion. This extension should be observed from the sagittal plane (Table 1, entry E). Exercises that facilitate triple extension should be used to train a powerful drive, such as wall knee drives, sled pushes, or single leg landmine drives (Table 2, entry D), as well as standard hip hinge movements such as glute bridges and deadlifts. If the pitcher struggles with achieving this triple extension position, an assessment of hip flexor muscle length, such as the modified Thomas test may be useful.^31^ Tightness in the iliopsoas may contribute to an excessive anterior pelvic tilt and subsequently hip flexion. Continued difficulty may also suggest the need for improved lumbopelvic stabilization and dissociation. A recommended exercise is standing rapid hip extensions, where athletes are encouraged to maintain a neutral pelvis position, quadruped hip extension, and a neutral lumbar spine (Table 2, entry D).

#### Fault: Early Rotation Toward Pitching Arm

During the stride, the upper body and hips should continue to remain square toward home plate to promote force generation in the direction of home plate. Although more evidence is needed to determine the exact inhibition of early trunk rotation toward the pitching arm, previous literature suggests that force generation needs to remain in line with home plate for optimal pitch velocity (Table 1, entry E).^26^

#### Fault: Failure to Land With Foot Angled Near 45°

At SFC, the stride foot should be rotated between 0 and 45° toward the pitching arm side (Table 1, entry F). Stride foot rotation can be assessed by measuring the foot angle, as mentioned above at SFC.^11^ Drawing a direct line toward home plate from the middle of the pitching rubber can help pitchers visualize proper foot placement. Excessive stride foot rotation toward the pitching arm side at SFC may contribute to early hip opening toward the throwing arm during stride. Early hip rotation may cause over-rotation of the trunk and pelvis and may also place excessive rotational stress on the knee.^29^ Conversely, a stride foot angle more toward home plate at SFC limits hip rotation and trunk rotation toward the pitching arm, which can also increase stress to the shoulder and result in a loss in pitch velocity.^29^ In addition, a stride foot angle positioned more toward home plate may also result in the pitching arm needing to adopt a different arm path to clear the hips during ball release and follow-through.^34^

#### Fault: Failure to Land With Stride Foot on the Power Line

Failure to land with the stride foot on the power line is another common fault.^29^ The power line represents an imaginary line drawn from the center of the pitching mound to the center of home plate. When the pitcher lands with the stride foot closer to the pitching arm side, there is a greater propensity for early and excessive trunk rotation toward the pitching arm side. Conversely, landing with your foot too far toward the glove arm side can limit the amount of trunk rotation available for energy transfer.^29^ A classic drawing of a power line in the dirt, along with verbal cues, can assist a pitcher in achieving an optimal landing position.

#### Fault: Oblique Arm Circle Path

It is also important for the pitcher to keep their pitching arm in a relatively straight path toward home plate. It is important to start the arm circle on a straight path early in the wind-up. A wide throwing arm backswing (shoulder horizontal abduction) during the wind-up may cause the arm to cross the body’s midline during the early stride, disrupting the path of the arm circle. If the arm crosses the body’s midline in early stride, inertia promotes continuation of the arm along this altered path leading to shoulder horizontal abduction through late stride and acceleration phases. Arm position can be assessed by looking down the power line from a posterior view (Table 1, entry G). A pitcher’s throwing arm should not horizontally adduct across the midline and should remain relatively perpendicular to the ground through the duration of the arm circle. Trunk and scapular stability are critical to creating a stable base for shoulder rotation. Upward scapular rotation is also important to position the glenoid for abduction range of motion during the pitch. Scapular stabilization exercises are recommended to improve the pitcher’s ability to maintain proper arm position. Exercises such as banded Ws, suspended rows, and Y lift-offs are recommended to strengthen the middle and lower trapezius muscles. In addition, closed chain punches are recommended to strengthen the serratus anterior muscle (Table 2, entry E). Trunk and scapular stability can be assessed clinically with the closed kinetic chain upper extremity stability test.^10^

#### Fault: Front Side Collapse at SFC

The trunk, hip, and knee should remain in a relatively extended position at SFC to create a stable base for optimal energy transfer. Excessive collapse (indicated by trunk flexion, hip flexion, and/or knee flexion) at SFC may disrupt efficient energy flow through the kinetic chain.^28^ Sagittal alignment can be assessed at SFC by drawing a vertical line from the head to the ground, which should pass through the umbilicus (Table 1, entry H). Failure to maintain an extended position at SFC may be caused by weakness in the gluteal muscles, other musculature of the core, or the quadriceps. Exercises to strengthen these muscle groups such as hip thrusters, planks, and single leg squats, are recommended (Table 2, entry F).

#### Fault: Stride Knee Valgus at SFC

The stride knee should be maintained in a neutral position at SFC given knee valgus can place excessive stress on the knee joint. To assess optimal frontal plane knee alignment, a vertical line can be drawn from the knee joint center down to the ground. If the line does not pass through the ankle joint center, the knee is in a poor position (Table 1, entry I). As previously mentioned, neuromuscular control training can be used to address poor frontal plane knee alignment (Table 2, entry G).

#### Fault: Excessive Trunk Lean at SFC

The trunk should also be controlled at SFC with minimal lean or tilt in any direction.^40,45^ The pelvis and trunk should be rotated toward the pitching arm side (Table 1, entry J); however, excessive trunk and pelvis rotation can limit kinetic chain sequencing and place undue stress on the shoulder and elbow.^45^ As mentioned above, lumbopelvic stabilization exercises and trunk proprioceptive training can address too much pelvis rotation or improper trunk alignment. Pitchers should demonstrate sufficient pelvis and trunk rotation or dissociation. Otherwise, a measurement of thoracic spine mobility may be warranted in which case the lumbar locked thoracic rotation test can be used.^16^ Although there is a lack of literature surrounding the ideal rotation for the softball pitcher, baseball pitchers are encouraged to demonstrate ≥50 degrees of rotation in each direction.^17^ Thus, it is recommended that softball pitchers aim to achieve a similar degree of rotation. Poor spinal range of motion can be addressed with thoracic mobility exercises, such as threading the needle or book openers performed in the side lying or half kneeling position (Table 2, entry H).

### Acceleration Phase

The acceleration phase begins at SFC and ends at ball release with the throwing arm experiencing the highest forces at the shoulder.^3,60^ The body lands back in a closed kinetic chain position, where both feet are in contact with the ground at SFC. Meanwhile, the latissimus dorsi, pectoralis, and serratus anterior adduct the throwing shoulder and the elbow extends as the ball moves through the back half of the arm circle and prepares for ball release.^19,37^ The kinetic chain theory states that energy should flow from lower body proximal segments to upper body distal segments in succession.^6^ Therefore, the lower body largely initiates the movement during the wind-up and stride phase but also provides a stable base upon which the upper body can rapidly transfer energy until the ball is released during the acceleration phase.^1,4^ During this acceleration phase, the drive leg is dragging behind the pitcher to close the gap between the planted stride leg. Ideally, only the inside of the big toe on the drive leg should touch the ground (Table 1, entry H); however, much variation exists. The drive hip should leave room for the throwing arm to pass but should also stay close to the power line to additionally aid in generating momentum toward home plate.

### Assessment and Training Strategies

#### Fault: Arm Circle Path Not Perpendicular to the Ground

The acceleration phase reaps the reward of proper body positioning during the wind-up and stride phases. For example, if the arm circle is initiated along a path perpendicular to the ground, it is likely to remain in that slot during this short and quick phase. Keeping the arm circle close along the power line ensures no additional or extraneous movement detracts from acceleration during this phase (Table 1, entry K). Failure to keep the arm in a slot close to the body may be caused by early and excessive trunk rotation toward home plate. This can result in the body blocking the arm’s path and causing the pitcher to bring the arm into abduction to clear the body and deliver the pitch.

#### Fault: Poor Trunk Control

Adequate positioning and neuromuscular control of the trunk is essential to ensure proper timing of trunk rotation and strategies suggested earlier to improve trunk proprioception are recommended. The pectoralis and serratus muscles are active in this phase to adduct the shoulder, so they should also be strengthened.^37^ Exercises such as push-ups, closed chain serratus punches, and serratus floor slides are suggested (Table 2, entry I). The push-up test can also be used clinically to assess a pitcher’s upper body strength.^29^

#### Fault: Drive Leg Straying From the Power Line

Keeping the drive leg close to the power line is ideal during the drag. A commonly observed fault involves the pitcher allowing their back leg to drag too far to the glove arm side of the power line. This widened drag takes the pitcher off the power line and can impede their trunk position, specifically causing excessive trunk flexion and lessening their ability to rotate trunk and hips toward home plate through acceleration. This can be assessed with a frontal plane view of the power line (Table 1, entry K). Poor transverse plane control of the pelvis may be a contributing factor to this fault. Thus, single leg neuromuscular training strategies that involve transverse pelvis plane control are recommended, such as anti-rotation single leg squats or rotational hops (Table 2, entry J).

### Follow-Through Phase

The follow-through is the final phase of the pitch that occurs from ball release through to either the end of the motion or a specific number of milliseconds (typically 10 ms) after ball release.^45^ Adequate slowing down of segments is important to safely dissipate the high pitching-arm forces generated during the high-velocity acceleration phase. Maintaining balance and stability over the stride leg while undergoing rapid changes in trunk position and upper body segments is important to decelerate from the pitch effectively. Maintaining a stable foundation through the follow-through via balance on the stride leg can also aid in a pitchers’ accuracy and control when delivering the pitch. Remaining balanced after the pitch is also important to help pitchers position themselves to play defense after ball release.

### Assessment and Training Strategies

#### Fault: Early Rotation Toward Pitching Arm

During the follow-through phase, the pitcher must effectively slow the arm, which requires adequate eccentric strength of the posterior shoulder musculature. Eccentric strengthening of the posterior shoulder musculature is recommended, including exercises such as eccentric side lying external rotation, suspended eccentric Ys, and eccentric banded horizontal abduction (Table 2, entry K). Whereas upper body eccentric strength is needed to support the follow-through, lower body balance is also necessary. The pitcher must maintain proper single leg balance on the stride limb while the trunk rotates toward the glove side. This can be assessed with a frontal plane view by drawing a line from the knee joint center down to the ground and ideally passing through the ankle joint center (Table 1, entry L). The optimal position is observed when the line is roughly in line with the ankle joint center, and suboptimal alignment is observed when the line falls outside the ankle joint center toward the glove arm side. The single leg squat test can be used to clinically assess single leg stability. Exercises such as single leg squats and single leg Romanian deadlifts with trunk rotation are recommended (Table 2, entry L).

## Biomechanical Pitch Type Considerations

Softball pitchers have an arsenal of pitch types used to deceive batters. The most common pitch types include the fastball, change-up, rise, curveball, drop ball, and screw ball.^13^ Pitchers may also opt to throw some combination of 2 pitch types (ie, drop-curve). To date, most biomechanical studies analyzing mechanics are conducted with pitchers throwing only a single pitch type for analysis, such as the fastball.^3,27,44,59,60^ The extrapolation of results to other pitch types is limited as kinematic and kinetic differences have been documented between pitch types.^13,43^ It is important to understand how different pitches may elicit various biomechanics to obtain desired pitch outcomes and movement on the ball. Whereas various body positioning may be required for specific pitches, certain phases of the movement should remain relatively unchanged. For instance, the wind-up phase of the pitch should remain unchanged during different pitch types to conceal pitch type from the batter; however, the subsequent phases of the pitch may portray slight alterations per pitch type.^13^

Specific biomechanical comparisons show that collegiate softball pitchers experience more trunk flexion during the drop ball than the fastball.^13^ Also, during the later phases of the pitch, pitchers throwing a curveball demonstrated more posteriorly shifted center of mass compared with when they threw a drop ball.^13^ Trunk and center of mass positioning differences are expected considering the intended trajectory and pitch movements desired per pitch type. For example, drop ball performance requires a bit of forward trunk lean to aid the downward movement of the ball, while the rise ball requires slight trunk extension to aid the upward movement of the ball at release.

Stride length and direction are also known to change subtly depending on whether pitchers need to be on top of the ball at release (ie, drop ball—shortened stride) or behind and under the ball at release (ie, rise ball—lengthened stride). Similarly, pitches that move horizontally require the stride to change directions subtly. For example, curveballs require a stride positioned slightly more toward the pitching arm side of the power line. This SFC position creates a pivot point for the upper body to rotate about and helps elicit curve spin on the ball (ie, spin perpendicular to the ground and in the direction of the glove arm side of the plate). Meanwhile, screw balls require a stride positioned slightly more toward the glove arm side of the power line. Screw ball execution requires this stride to create space for the arm to adduct toward the pitcher’s body during the back half of the arm circle. Then, as the pitch progresses, the pitching arm trajectory moves from being close to the body in adduction, to away from the body in abduction near ball release and through follow-through. This slightly abnormal arm circle helps to elicit ball flight movement in the direction of the pitching arm side of the plate. Similarly, and for other pitch types, if a pitcher desires to throw to the right side of the plate, they tend to step a bit more in that direction, and vice versa for throwing toward the left side of the plate.

From a kinetics perspective, the change-up has significantly less peak elbow anterior force and peak shoulder distraction force during the acceleration phase of the pitch compared with the fastball and curveball.^43^ The decreased upper extremity kinetics found in the change-up are likely due to the decreased pitch speed. Although little is known regarding the effect of pitch type on the presence of pain and overuse injury, understanding the differing demands placed on the body between pitch types is important for pain and injury etiology and workload management for pitchers overall.

## Energy Flow

Efficient proximal to distal energy flow through the kinetic chain during softball pitching may maximize performance and reduce stress on the upper extremity.^39,45,46^ Specifically, energy generated by the lower extremities and lumbopelvic hip complex (region of the pelvis and trunk) is postulated to be transferred to the humerus, forearm, and finally hand immediately before ball release.^7,35,45,46^ Therefore, energy flow terminology is used frequently in softball pitching injury prevention and performance enhancement studies.^28,45,46,58^ Although most existing biomechanical analyses have focused on individual kinematic and kinetic parameters, emerging studies have investigated energy flow during softball pitching.^4,51^ Several factors, including clinical range of motion, strength parameters, and mechanics may alter energy flow through the kinetic chain. Oliver et al^51^ were the first to examine the relationship between energy flow, performance, and clinical measures of strength and range of motion in a collegiate softball pitching population. Greater drive hip external rotation strength relates to increased net energy flowing out of the distal ends of the trunk and humerus. Further, higher peak energy rates flowing out of the distal end of the trunk (the end closest to the shoulder) and pitching-arm segments were associated with greater pitch velocities.^51^ The results of Oliver et al^51^ support the importance of monitoring hip strength in softball pitchers since muscular weakness is related to decreased energy flow through the kinetic chain and may negatively impact pitch performance.

Considering softball pitchers experience a high prevalence of upper extremity pain, several studies have examined the difference in kinematics and kinetics between pitchers with and without upper extremity pain.^40,45^ Specifically, the results showed softball pitchers with upper extremity pain presented with altered trunk kinematics and higher shoulder distraction forces.^40,45^ Altered mechanics may disrupt energy flow through the proximal kinetic chain resulting in undue stress being placed on the upper extremity. Therefore, to test this postulation, Bordelon et al^4^ investigated the difference in pelvis and trunk energy flow between collegiate softball pitchers with and without upper extremity pain. The analysis determined no significant differences between pelvis and trunk energy flow and pitch velocity between pitchers with and without upper extremity pain. In conjunction with the findings from previous research, altered pitching mechanics may serve as compensation to maintain proximal energy flow and performance.^4^

Overall, greater energy flow through the trunk and upper extremity during softball pitching is related to higher hip external rotation strength and pitch velocities.^51^ Further, pitchers with upper extremity pain may alter their mechanics to maintain performance and proximal energy flow patterns.^4^ Although the aforementioned studies have provided valuable insight to understanding of energy flow through the kinetic chain during softball pitching, they were limited to segment power analyses. A more in-depth analysis of energy generation, absorption, and transfer is warranted to fully understand how patterns might be related to pitching mechanics, performance, and stress placed on the upper extremity.

## Functional Characteristics

The softball pitch is a total body dynamic movement, where proximal stability is essential for distal mobility to enhance pitch velocity and reduce the risk of upper extremity injury. Muscular weakness, instability, or inadequate range of motion in the proximal lower extremities is postulated to disrupt energy flow through the kinetic chain. These disruptions result in decreased energy generated by the lower extremities and lumbopelvic-hip complex and place undue stress on the upper extremity to maintain the same level of performance. Due to the repetitive nature of the pitch, this may place softball pitchers at increased risk of upper extremity pain and overuse injury.^32^ Therefore, it is essential to have functional assessments to identify areas of muscular weakness, instability, and inadequate range of motion when developing injury prevention and performance enhancement strategies.

Oliver et al^46^ investigated the differences in functional characteristics between collegiate softball pitchers with and without upper extremity pain. Pitchers with upper extremity pain had decreased throwing side hip external rotation range of motion, throwing side hip internal rotation isometric strength, glove side hip external rotation isometric strength, bilateral shoulder external rotation isometric strength, and glove side shoulder internal rotation isometric strength compared with pitchers without pain. Overall, the results showed pitchers with upper extremity pain had inadequate hip range of motion and muscular weakness at the shoulder and hip. Further, Downs Talmage et al^14^ also examined the range of motion adaptations across a simulated game in softball pitchers. Throwing shoulder internal rotation range of motion, glove arm shoulder internal rotation and external rotation range of motion, stride hip external rotation range of motion, and drive hip internal and external rotation range of motion significantly decreased across a simulated game.^12^ Therefore, injury prevention efforts include monitoring hip and shoulder rotation range of motion as well as isometric strength in softball pitchers. These assessments should include monitoring before and after games to monitor acute adaptations and recovery for individual pitchers.

A common exercise used to assess lower extremity and lumbopelvic-hip complex stability in softball pitchers is the single leg squat.^2,5,28^ Compensations observed in the single leg squat such as knee valgus, trunk rotation, and excessive trunk flexion are attributed to gluteal muscle weakness.^8,53,56,57^ This muscle group is important for stabilizing the trunk during single leg support, which is necessary for efficient function of the kinetic chain during the softball pitch. This is supported by Friesen et al,^28^ who determined compensations during the single leg squat were associated with pathomechanics during the softball pitch. Specifically, greater trunk rotation and flexion at the bottom of the single leg squat were related to increased knee valgus and trunk flexion at foot contact during the pitch. The findings support using the single leg squat to identify lumbopelvic-hip complex instability that may contribute to pathomechanics during the softball pitch.

## Anthropometrics

The importance of understanding body anthropometrics, shape, and size has come under recent evaluation in considering softball pitch biomechanics, injury risk, and performance. Pitchers can be of all shapes and sizes given the sport allows for success despite specific anthropometrics. Although success is not limited to pitchers of specific body types, recent literature acknowledges some findings associated with pitchers who possess excess body fat compared with those with a healthy body fat percentage. Assessment of body anthropometrics is important given pitchers tend to increase body fat throughout a season as opposed to other position players,^52^ and typically present the highest body fat percentages on collegiate softball teams.^9^

Although increased mass may theoretically benefit performance, recent research is contradictory. For instance, body mass index and fat free mass index are positively associated with pitch velocity whereas body fat mass and fat mass index are negatively associated with pitch velocity.^26^ Thus, body mass does not seem to directly contribute to better performance and research indicates that increased body mass is common among pitchers in pain.^32,40^

Differences are also noted biomechanically. Both kinematics and kinetics vary according to body fat percentage,^19,24,25^ suggesting body fat mass may influence kinetic chain sequencing and injury propensity.^18,22^ Further, asymmetry of functional characteristics exists between body fat percentage groups,^20,23^ indicating that body fat mass can alter movement and contribute to various biomechanical compensations during the softball pitch.

Current research acknowledges differences considering body fat and efforts are made to improve the overall understanding of body fat implications on general athlete health and pitching success.^18-20,22-25^

## Conclusion

The windmill softball pitch has long been considered natural given the underhand nature of the pitch. Still, research highlights the vast implications of body kinematics, kinetics, anthropometrics, energy flow, and functional characteristics on softball pitchers’ health and performance.

Pitching is a complex motor skill with a high risk of overuse injury at both the youth and collegiate levels. Proper biomechanics are essential to minimize injury risk and optimize skill performance. Research has shown that a pitchers’ functional characteristics like shoulder, hip, trunk, and pelvis range of motion and strength may affect kinematics and ball velocity. Athlete anthropometrics also likely contribute to injury risk.

## Supplemental Material

sj-docx-1-sph-10.1177_19417381251323610 – Supplemental material for Biomechanics of Fastpitch Softball Pitching: A Practitioner’s Guide Supplemental material, sj-docx-1-sph-10.1177_19417381251323610 for Biomechanics of Fastpitch Softball Pitching: A Practitioner’s Guide by Kenzie B. Friesen, Lauren S. Butler, Nicole M. Bordelon, Jessica L. Downs-Talmage, Glenn S. Fleisig, Sophia Ulman and Gretchen D. Oliver in Sports Health
