# Supplementary material for: Biomechanics of Fastpitch Softball Pitching: A Practitioner’s Guide
Source: Sports Health. 2025 Apr 3;17(6):1200–13. doi: 10.1177/19417381251323610 (PMC11969493; doi:10.1177/19417381251323610)
Supplement: sj-docx-1-sph-10.1177_19417381251323610 – Supplemental material for Biomechanics of Fastpitch Softball Pitching: A Practitioner’s Guide [file sj-docx-1-sph-10.1177_19417381251323610.docx]

| Table 2A: Quick drop to split stance drills |
| --- |
| Table 2 A: Rapid tempo rear foot elevated split squats |
| Table 2 A: Split stance medicine ball overhead slams |
| Table 2 B: Banded trunk single leg squats |
| Table 2 B: Single leg bridges |
| Table 2 B: Super clams |
| Table 2 C: Knee banded plyometric training |
| Table 2 C: Knee banded single leg squats |
| Table 2 C: Lateral step downs |
| Table 2 D: Landmines |
| Table 2 D: Quad hip extension |
| Table 2 D: Sled pushes |
| Table 2 D: Standing rapid hip extensions |
| Table 2 D: Wall knee drives |
| Table 2 E: Banded Ws |
| Table 2 E: Serratus punches |
| Table 2 E: Suspended rows |
| Table 2 E: Y lift-offs |
| Table 2 F: Hip thrusters |
| Table 2 F: Planks |
| Table 2 F: Single leg squats |
| Table 2 H: Book openers |
| Table 2 H: Thread the needle |
| Table 2 I: Pushups |
| Table 2 I: Serratus floor slides |
| Table 2 J: Anti-rotation single leg squats |
| Table 2 J: Rotational hops |
| Table 2 K: Eccentric side lying external rotation |
| Table 2 K: Eccentric banded horizontal abduction |
| Table 2 K: Suspended eccentric Y's |
| Table 2 L: Single leg Romanian deadlift with trunk rotation |

**Biomechanics of Fastpitch Softball Pitching: A Practitioner’s Guide**

Legend of Supplemental Videos, companion to Table 2
